# Brief psychotic episode in an adult without medical antecedents after suffering the indirect consequences of the Russian-Ukrainian war

**DOI:** 10.1192/j.eurpsy.2023.1929

**Published:** 2023-07-19

**Authors:** R. G. Troyano, M. Fariña Francia, E. Marimon Muñoz, I. Fernandez Marquez, E. Miranda Ruiz, M. Arroyo Ucar, J. Ramirez Gonzalez, S. Ferreiro Gonzalez, C. Hidalgo, A. Quispe

**Affiliations:** Psychiatry, Hospital Consorci Sanitari de Terrassa, Terrassa, Spain

## Abstract

**Introduction:**

Almost nine months after the start of the war between Russia and Ukraine, millions of people have been affected physically, economically and mainly mentally. Those who have stayed in their homeland, and the ones that have chosen to emigrate to a safer place.

**Objectives:**

The objective of this article is to assess the importance of social stressors in the onset of a brief psychotic episode, even in the absence of substance abuse or previous illnesses.

**Methods:**

The case of a 45-year-old woman is described, known by the Pediatric Emergency Service, for being the tutor of a patient who suffered from anxiety attacks, having emigrated without her parents from Ukraine together with her 5 brothers. The psychotic episode begins when our patient gets notified that she must abandon the custody of the girl, because she will have to go to Turkey with her legal guardians. The family explains the behavioral changes that the patient made and how the clinical picture worsened.

**Results:**

She was admitted at the Hospital’s Psychiatry Service and antipsychotics treatment started. After 5 days, the episode had completely been solved.

**Conclusions:**

In conclusion, we highlight the importance of social problems in the development of a psychiatric pathology and the necessary elements to prevent it: family support network, fast and efficient care services and availability of hospital and pharmaceutical resources.

**Disclosure of Interest:**

None Declared

